# Precipitation within localised chromium-enriched regions in a Type 316H austenitic stainless steel

**DOI:** 10.1007/s10853-017-1748-4

**Published:** 2018-01-09

**Authors:** A. D. Warren, I. J. Griffiths, P. E. J. Flewitt

**Affiliations:** 1 Interface Analysis Centre, HH Wills Laboratory, University of Bristol, Bristol, BS8 1FD UK; 2 School of Physics, HH Wills Laboratory, University of Bristol, Bristol, BS8 1FD UK

## Abstract

A Type 316H austenitic stainless steel component containing Cr and impurity element-rich localised regions arising from component fabrication was aged for a prolonged period during service at a temperature of approximately 550 °C. These regions make up approximately 5% of the total volume of the microstructure. Previous work has shown that these regions contain ferrite and carbide precipitates and a finer austenite grain size than the adjacent matrix. The present study has used high-resolution transmission electron microscopy combined with compositional microanalysis to show that these regions have a highly complex microstructure containing G phase, chi phase and intragranular γ′ precipitates within the austenite grains. There is phosphorus migration to the chi austenite phase boundary, and the basis for this equilibrium impurity segregation is discussed. A Cr-depleted region was observed surrounding the chi phase precipitates, and the impact of this on the other precipitates is considered. The diversity of precipitates in these Cr-rich regions means that they behave significantly differently to the bulk material under long-term creep conditions leading to preferred nucleation and growth of creep cavities and the formation of localised creep cracks during service.

## Introduction

The microstructure of an austenitic stainless steel has a significant role in controlling the physical, chemical and mechanical properties. In the case of high temperature, ~ 550 °C, nuclear electrical power generating plant subject to extended periods of operating, > 10^5^ h, changes in the microstructure can have a significant influence on the overall service life. For example, the presence of secondary phase precipitates, such as ferrite, has the potential to significantly alter the creep properties by changing the behaviour of creep damage accumulation [1–5]. The evolution of secondary phases during thermal ageing of Type 316 austenitic stainless steels has been observed experimentally [5–9] and predicted thermodynamically [10], driven by a favourable Gibbs energy [11]. These precipitates will nucleate at discrete sites within the overall microstructure and grow over time. Austenitic stainless steels are known to form a wide range of precipitates depending on the specific composition and thermo-mechanical history. Indeed, up to 18 different precipitate types have been variously identified after prolonged ageing [7], leading to highly complex microstructures. Precipitates commonly found include both α- and δ-ferrite, various carbides [8], and complex phases such as sigma phase, R phase and G phase. One of the less encountered precipitates is G phase, a silicon containing FCC intermetallic phase (*a* = 1.115–1.120 nm [12]) with a nominal composition observed in austenitic stainless steels [12–20] of (Ni/Fe/Cr)_16_(Nb/Ti)_6_Si_6_ [12]. By comparison, in duplex stainless steels [12, 21, 22] the composition is (Fe/Ni)_16_(Mn/Cr)_6_Si_7_ [19]. Other studies have found that substitution of Mo for Ni is possible [14, 23]. G phase precipitates form within the ferrite regions of duplex [21, 22] and nominally austenitic stainless steels (e.g. Type 300 series austenitic steels) [13, 15, 16, 19] during ageing within the temperature range of 250–500 °C [12, 14, 22]. This phase typically nucleates and grows at austenite grain boundaries [21, 24], and/or at austenite–α-ferrite phase boundaries but in the latter case it has a cube-on-cube orientation relationship with the ferrite [19, 21]. G phase evolution in austenite is associated with exposure to higher temperatures, within a nominal range of 500–800 °C [13], where the kinetics have been purported to be controlled by the rate of Si diffusion [21]. Lower ageing temperatures in austenitic stainless steels favour austenite grain boundary precipitation of G phase [12, 13], whilst intragranular precipitation is observed at higher temperatures [12]. Steels with high volume fractions of G phase have been found to be embrittled [19, 25] when subjected to room temperature fracture, and as such the phase promotes intergranular fracture.

Chi phase is a BCC intermetallic phase (*a* = 0.881–0.895 nm [12]) the precipitation of which only occurs in Mo- or Ti-containing stainless steels [12, 26]. Depending on the relative concentrations of Mo and Ti, the composition of the chi phase ranges from (Fe/Ni)_36_Cr_12_Mo_10_ to (Fe/Ni)_36_Cr_12_Mo_3_Ti_7_ [12, 24, 26–28], and there is potential for substitution of other elements [28]. The composition of chi phase is frequently likened to that of sigma phase, with the principle differences being the solubility of carbon [12, 26] and higher concentrations of molybdenum [26, 29, 30]. Chi phase has been observed to nucleate and grow preferentially into the ferrite regions of dual phase (austenite-ferrite) steels [26, 29–32], at grain boundaries of δ-ferrite [26, 32], at austenite–δ-ferrite grain edges and nodes [32] and austenite–δ-ferrite phase boundaries [29, 30, 32]. This preference has been linked to the greater concentrations of Cr and Mo present in ferrite, and the higher rate of diffusion of these BCC elements through the ferrite (BCC) as opposed to the austenite (FCC) [31]. In addition, precipitation of chi phase has been observed associated with M_23_C_6_ carbide precipitates [33]. The presence of chi phase precipitates in general decreases room temperature fracture toughness and reduces corrosion resistance [12, 32], although it has been observed to not significantly modify creep deformation or damage accumulation behaviour of austenitic stainless steel [27, 34].

The degree of deformation (reduction in cross section) which occurs during the fabrication of a component can have a significant role in the evolution of subsequent microstructure. Highly worked thin cross-sectional components, such as tubes, tend to be subject to more severe deformation than thicker walled components such as headers. More severe deformation removes heterogeneous casting features arising from the original dendritic structure of the cast ingot [35]. The presence of localised elementally enriched regions in the microstructure can have a significant impact on subsequent precipitation within the component, as most secondary phases are composition dependant [12, 24]. When subject to smaller amounts of fabrication, deformation localised regions with a significantly different composition can remain. The consequences are that these localised variations in composition lead to an austenitic stainless steel with a non-uniform and complex microstructure when subjected to the long-term ageing typically encountered during service.

In addition to fabrication effects, precipitation is significantly influenced by cast-to-cast variation. Steel for plant construction will be supplied to a given specification, which consists of a range of acceptable compositions for specified elements. As such notionally identical components made of “Type 316H” steel may have different specific compositions. This can be further influenced by the fabrication effects already discussed. Outside of the monitored elements, there may also be disparities in trace impurity levels between both casts and manufacturers. The net effect of these variations in composition is that they can lead to differences in secondary phase precipitation. Thus, two components of different casts aged for the same time at the same temperature can have differences in the phases evolved and the volume fraction of these phases.

In this paper, we describe the microstructure of fine-grained, Cr-enriched regions arising as a consequence of failure to homogenise the original cast microstructure during the thermo-mechanical fabrication of an ex-service Type 316 austenitic stainless steel component subject to a post-service heat treatment. The heterogeneous microstructure is described together with the influence on the subsequent precipitation. The precipitates have been characterised using high spatial resolution electron optical techniques combined with STEM-EDX microanalysis. The precipitates identified are discussed with respect to the thermodynamics and kinetics of precipitation.

## Materials and methods

**Figure 1:**
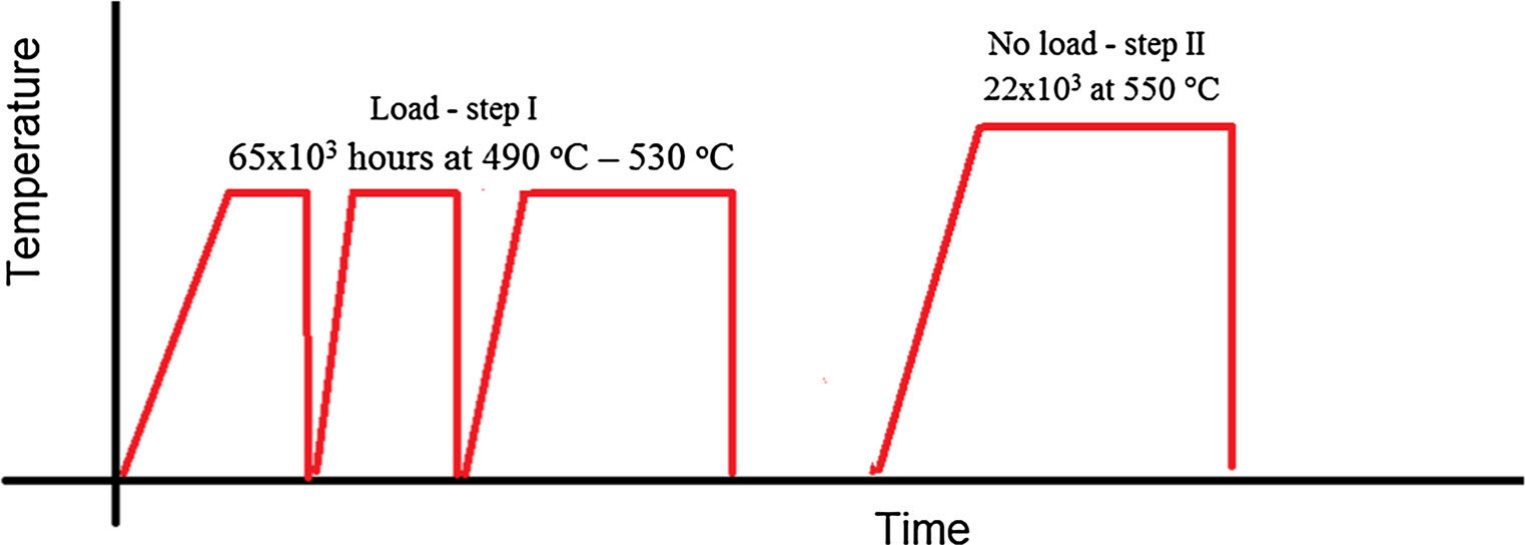
Schematic diagram showing the thermo-mechanical history of the specimen. The outages in step (I) are representative and as such are not to scale. Note the horizontal axis intersects at room temperature. After Warren et al. [5]

The material sample studied was 25 mm × 10 mm × 10 mm of Type 316H austenitic stainless steel, cut from the attachment weldment region of an advanced gas-cooled reactor (AGR) boiler header (provided by EDF Energy Ltd.). During service, the specimen experienced 65015-h operation in the temperature range 490–530 °C, followed by a post-service laboratory heat treatment of 22100 h at 550 °C to simulate the effects of further ageing. The post-manufacture thermo-mechanical history of the specimen is shown schematically in Fig. 1, and the composition of the specimen is given in Table 1. During manufacture, the material was solution treated for 3 h at 1050 °C, followed by a water quench. The material is known to contain approximately 0.2–0.5 vol% mixed δ- and α-ferrite following step (I) [5] which increased to approximately 2 vol% δ- and α-ferrite following the laboratory heat treatment, step (II) [5]. During service, the conditions were such that the specimen experienced creep, resulting in the formation of creep cavities and a significant creep crack [5].

**Table 1:** Composition of the ex-service Type 316H austenitic stainless steel header weldment (wt%), supplied by EDF Energy Ltd. following OES analysis. Error on measurements not supplied

C	Si	Mn	P	S	Cr	Mo	Ni	B	Co	N	Fe
0.06	0.4	1.98	0.021	0.014	17.17	2.19	11.83	0.005	0.10	–	66.23

Specimens were consecutively polished with silicon carbide papers and diamond pastes to obtain a 0.25-μm surface finish. Additional polishing using 0.1-μm colloidal silica was required to produce a suitably high-quality surface for electron backscatter diffraction to identify the fine-grained, Cr-enriched, residual casting regions [5], from which transmission electron microscopy (TEM) liftouts were prepared. Electron backscatter diffraction (EBSD) analysis was performed in a Zeiss SIGMA FEG-SEM, fitted with a high-speed camera (DigiView 3). All EBSD maps were collected with the SEM operating at 30 kV, and the specimen tilted by 70 degrees to the horizontal. Data were collected using orientation image mapping (OIM) software (Ametek, Utah, USA).

Liftouts were prepared from the specimen by gallium ion milling using a FEI Helios Nanolab 600i “Dual beam” SEM/FIB workstation. The liftouts were given a lower energy 5-kV ion mill at the end of the process to reduce the surface damage, which was confirmed by observation in the TEM [36]. Transmission electron microscopy (TEM) and scanning transmission electron microscopy (STEM) were performed using a JEOL-ARM 200CF with a Cs probe corrector, operating at 200 kV. Energy-dispersive X-ray spectroscopy (STEM-EDX) was performed using a 100-mm^2^ JEOL Centurion detector and NSS version 3.2 analysis software. Electron energy loss spectrometry measurements were made with a Gatan GIF Quantum 965 ER detector with the results process with the Digital Micrograph software.

## Results

**Figure 2:**
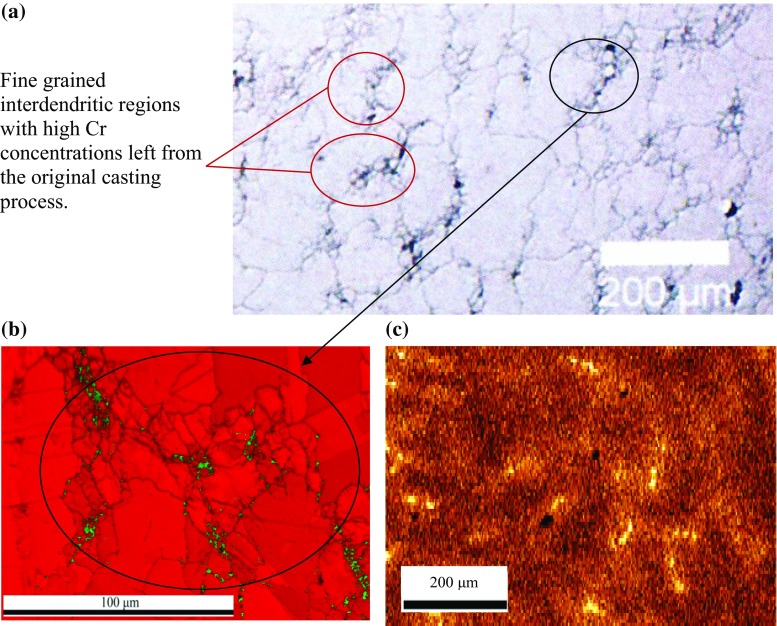
Characterisation of the ex-service Type 316H specimen. **a** A region from Chen et al’s optical micrograph, showing the distribution of fine-grained austenite/ferrite/M_23_C_6_ carbide regions [41], **b** EBSD phase map for a localised Cr-enriched region, with austentite in red and ferrite in green. **c** Cr Kα EDX map showing the distribution of Cr-enriched regions within a region

Previous studies on the ex-service Type 316H material by Chen et al. [37] and Warren et al. [5] identified localised regions with a fine grain size enriched in Cr, and impurity elements arising from the initial casting process [5, 37], Fig. 2a. The bulk of the microstructure comprises austenite grains with diameters of 20–100 µm [5], together with a distribution of approximately 2% by area ferrite and other intragranular precipitates [5]. Hence, subsequent fabrication, solution treatment and long-term thermal ageing had not fully removed the initial casting microstructure and the associated local composition fluctuations [5]. When the residual regions were investigated using EBSD, the grains contained a large number of small austenite grains (< 20 µm) with low angle sub-boundaries [5], and a much greater proportion of ferrite and chi phase precipitates than in the bulk of the material [5], Fig. 2b. The chromium-enriched regions can clearly be seen in the composition map, Fig. 2c.

**Figure 3:**
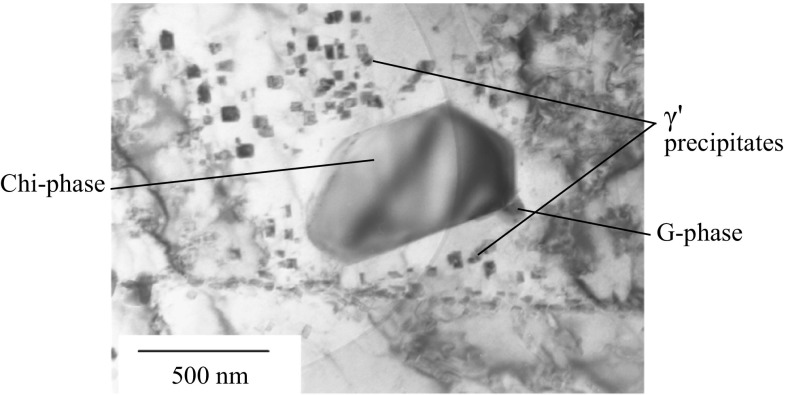
TEM bright-field image showing a γ′ precipitate-depleted region around a chi phase precipitate with associated G phase precipitate. The partial circle of different contrast on the left of the image is due to the relative positioning of the specimen with respect to the amorphous carbon support. Measurements for quantification were taken from precipitates sited over holes in the support grid

High-resolution TEM analysis of liftouts selected from the local high Cr regions revealed a range of precipitates. Compositions and lattice parameters for the bulk austenite and ferrite precipitates are summarised in Table 2. In many cases, the measured compositions of the phases show significant divergence from those given in previous studies on Type 316H austenitic stainless steels with a more uniform microstructure. A TEM bright-field image showing chi phase, G phase and γ′ is shown in Fig. 3.

**Table 2:** Measured average compositions, crystal structures and lattice parameters of the secondary phase precipitates present in the fine-grained, high Cr residual casting regions present in an ex-service + 22000-h aged Type 316H austenitic steel

Phase	Crystal structure	Measured lattice parameter (nm)	Mean measured composition—wt%										
			C	O	Al	Si	S	P	Cr	Mn	Fe	Ni	Mo
Austenite	FCC	0.358	–	–	–	3	–	–	8	2	66	10	–
α-ferrite	BCC	0.285–0.289	–	–	–	–	–	–	7 ± 0.7	^–^	84 ± 0.9	2 ± 0.1	–

C data excluded due to contamination; O and Al contributions are conflated with contributions from the grid and holder and thus excluded. Errors are the standard deviations

### Localised precipitation

In this section, we consider in detail each of the long-term ageing-induced precipitates identified in the 4 TEM liftouts cut from the localised Cr-enriched regions.

#### Chi (*χ*) phase

**Figure 4:**
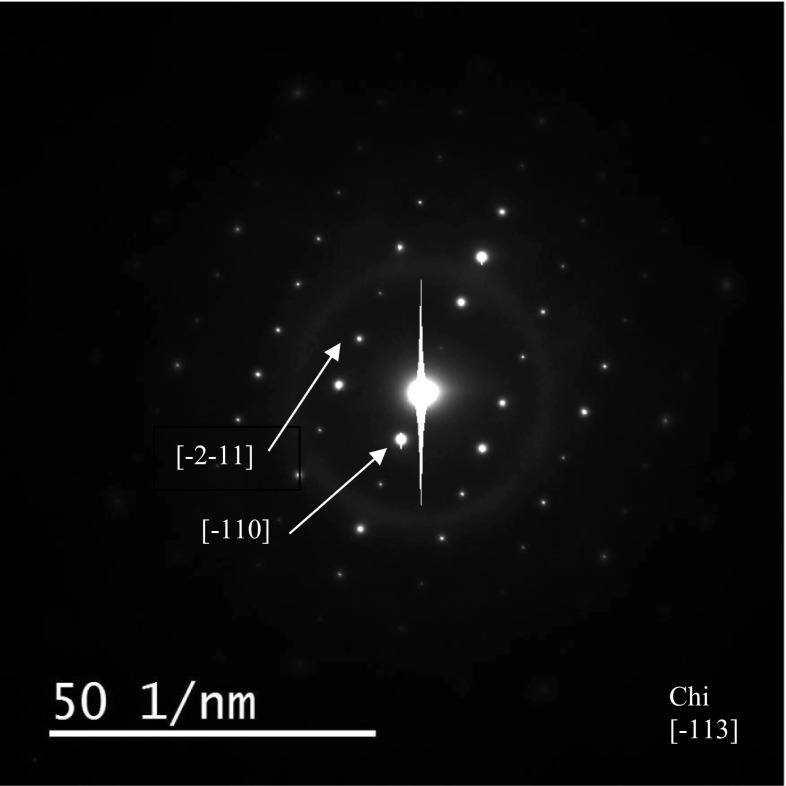
Selected area diffraction pattern observed from the chi phase, precipitate shown in Fig. 3

**Figure 5:**
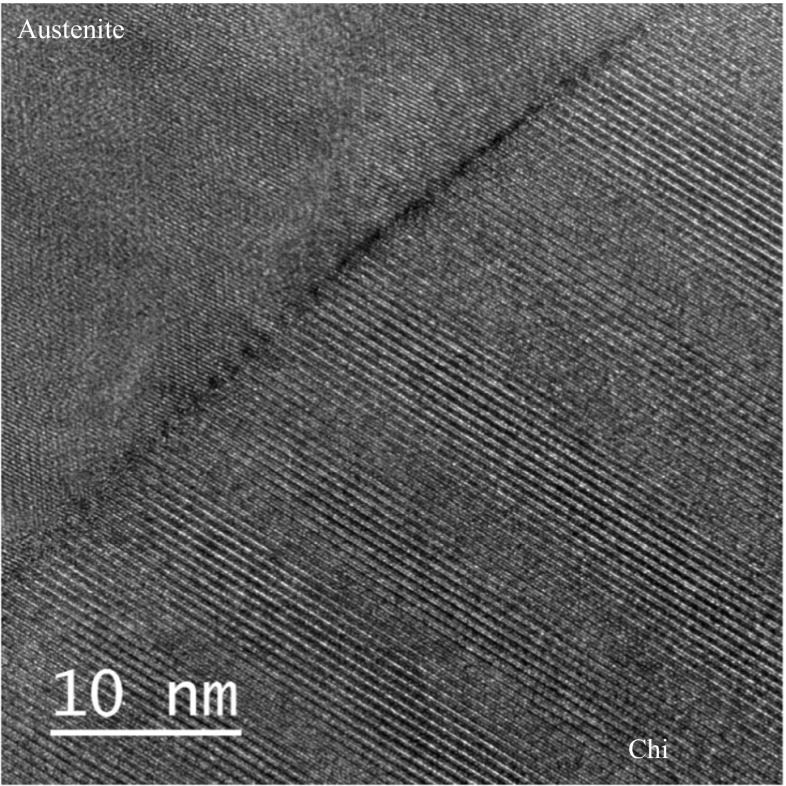
Atomic resolution TEM images of the chi phase (bottom right)–austenite (top left) interphase boundary in the ex-service + 22000-h aged Type 316H steel

Faceted precipitates of up to 1 μm equivalent diameter, at the austenite–austenite grain boundaries were identified as chi phase from the BCC crystal structure and lattice parameter (~ 0.8 nm), by electron diffraction. Figure 4 shows a typical chi phase [−113] electron diffraction pattern recorded from this specimen. An atomic resolution image, of an austenite–chi phase boundary is shown in Fig. 5 where the lattice spacing in the austenite is 0.17 nm, and the chi phase is 0.41 nm. The periodic variations in contrast along the interface are mismatch dislocations to accommodate lattice differences, and associated strain, between the two phases.

**Figure 6:**
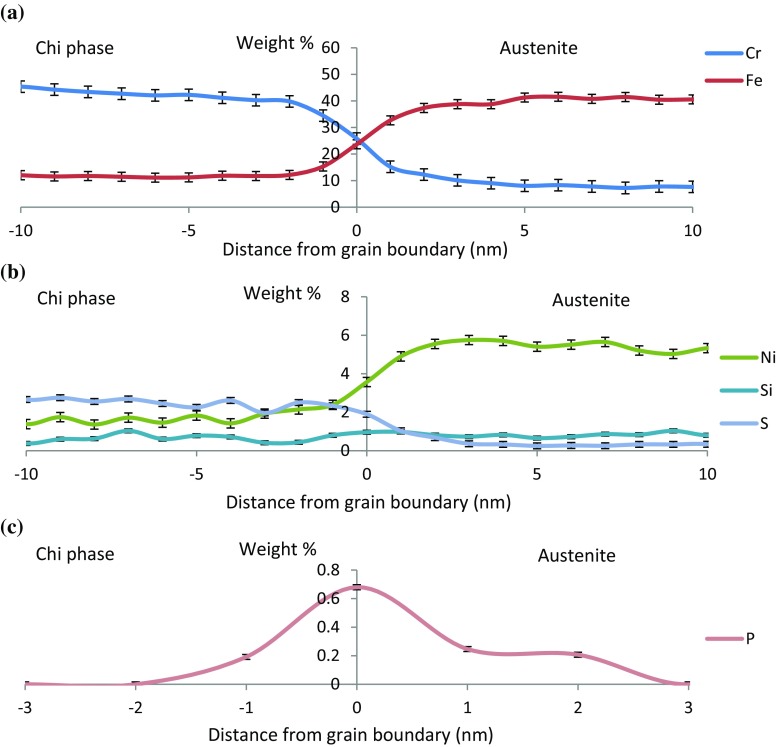
STEM-EDX line scans across an austenite–chi phase boundary. **a** The full range for Cr and Fe, **b** the low weight percentage elements, Ni, Si and S, **c** the uncorrected measurement of phosphorus enrichment at the interphase boundary. Errors are taken from the standard deviation of the data. Profiles were recorded at a dwell of 5 s per pixel, and 6 repeats, to give a total of 30 s per pixel

The composition of the chi phase precipitates was measured from seven precipitates using EDX microanalysis, giving an average measured composition of approximately (Fe/Ni)_15_Cr_26_Mo. This is a distinct difference compared with the composition reported in previous studies between (Fe/Ni)_36_Cr_12_Mo_10_ and (Fe/Ni)_36_Cr_12_Mo_3_Ti_7_ [12, 24, 26–28]. The composition measured at the austenite–chi phase boundary, Fig. 6a, shows the expected distribution of primary alloying elements (Cr, Fe) between the phases. In Fig. 6b, the lower concentration elements Ni, Si and S are shown. In particular, Si is associated with the chi phase. An increase was observed in the carbon signal due to deposition of carbon-containing contamination during the measurement, an increase with time rather than phase dependence. There is a distinct P peak of 0.68 wt% at the position of the phase boundary, Fig. 6c. The electron beam will be dispersed by the foil, so this value may be an underestimate of the true composition. However, the foil is < 100 nm and the incident electron probe is approximately 0.1 nm dia. so the extent of the beam dispersion will be small. In such cases, it is appropriate to adopt the simple correction to the measured concentration, *C*, given by Faulkner et al. [38] based on the more rigorous analysis proposed by Doig et al. [39]:

1 $$ C = C_{0} + \raise.5ex\hbox{$\scriptstyle 1$}\kern-.1em/ \kern-.15em\lower.25ex\hbox{$\scriptstyle 2$} \left( {C_{\text{b}} - C_{0} } \right)\left[ {{\text{erf}} \left( {\frac{{x_{d} + \raise.5ex\hbox{$\scriptstyle 1$}\kern-.1em/ \kern-.15em\lower.25ex\hbox{$\scriptstyle 2$} \,d_{0} }}{\sigma \sqrt 2 }} \right) - \,{\text{erf}}\left( {\frac{{x_{d} - \raise.5ex\hbox{$\scriptstyle 1$}\kern-.1em/ \kern-.15em\lower.25ex\hbox{$\scriptstyle 2$} \,d_{0} }}{\sigma \sqrt 2 }} \right)} \right] $$

where *C*_0_ is the solute concentration in the austenite matrix, *C*_b_ is the actual solute concentration at the boundary, *x*_d_ is the location of the beam relative to the boundary (using sub-pixel scanning means this can be approximated as zero as the beam will raster across the boundary within the relevant pixel), *d*_0_ is the width of the solute layer at the boundary (assumed as 1 nm due to equilibrium segregation) and *σ* the standard deviation of the electron probe distribution [39]:

2 $$ B = 2\left( { - 2\sigma^{2}  \ln 0.5} \right)^{\raise.5ex\hbox{$\scriptstyle 1$}\kern-.1em/ \kern-.15em\lower.25ex\hbox{$\scriptstyle 2$} } $$

where *B* is the electron probe diameter calculated using Eq. 1. Using this conversion factor gives the true concentration of phosphorus at the austenite–chi phase boundary as approximately 2.0 wt%. No corresponding change in any alloying or other impurity elements was observed.

#### G phase

**Figure 7:**
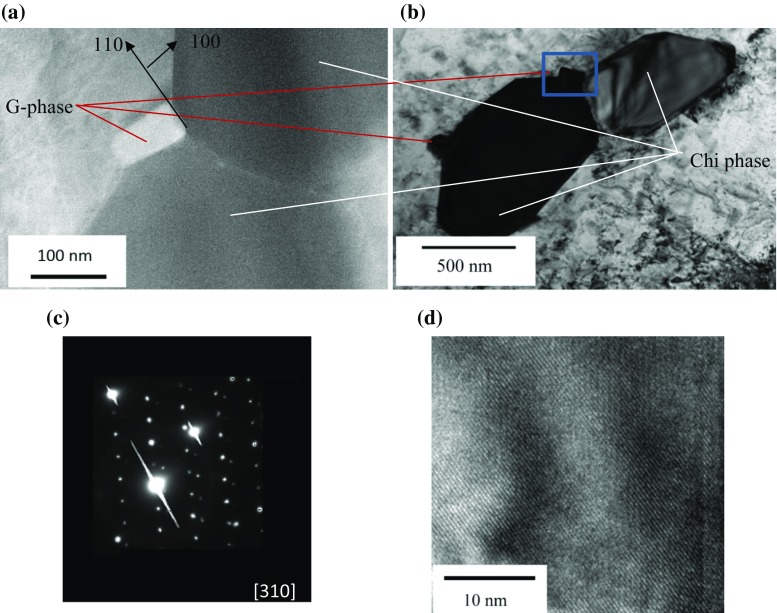
G phase precipitates associated with chi phase in the ex-service + 22000-h aged Type 316H austenitic stainless steel. **a** STEM image of G phase adjoining chi phase precipitates in an austenite matrix and **b** TEM image showing the close orientation relationship between the chi phase precipitate and its adjoining G phase precipitate in the austenite matrix. The blue box corresponds to the region in **a**. **c** Selected area diffraction pattern from a typical G phase precipitate, showing additional diffraction spots arising from matrix and chi phase precipitates. **d** atomic resolution STEM images of G phase crystal lattice

**Figure 8:**
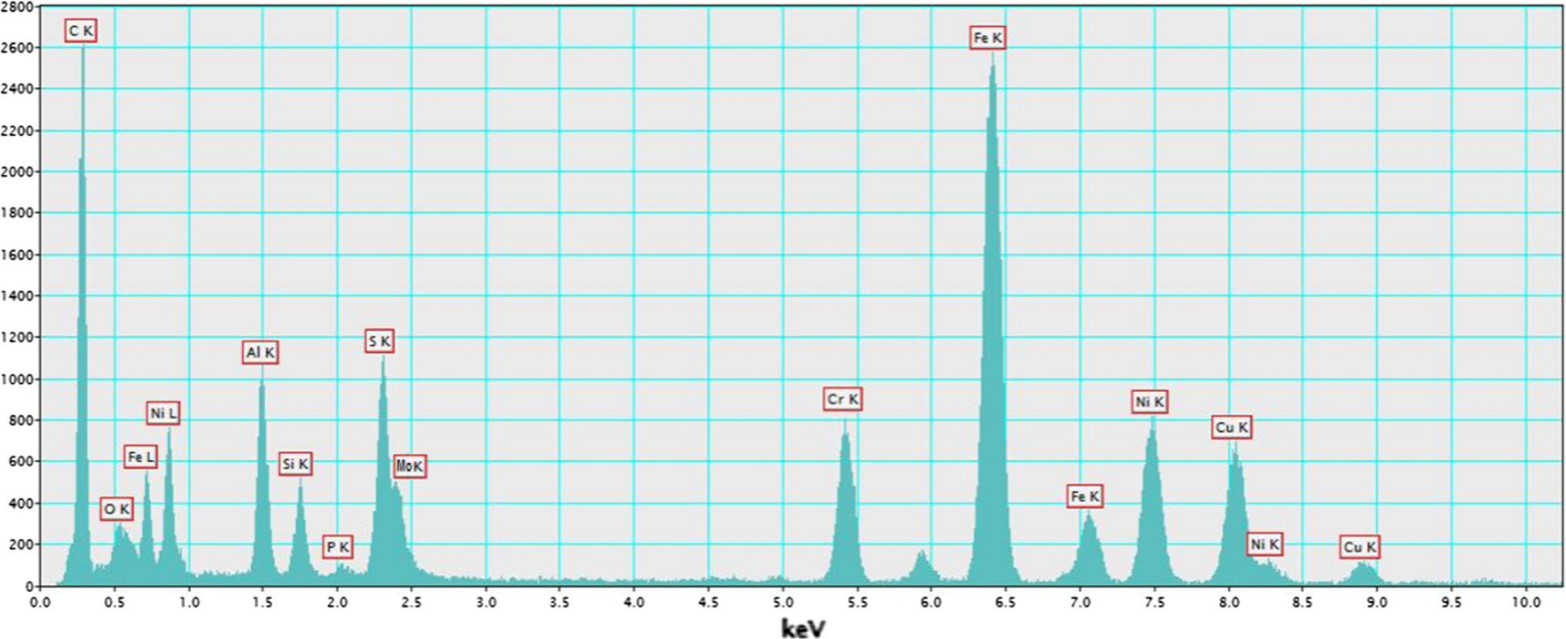
XEDS microanalysis spectrum of G phase precipitates, using STEM-EDX, showing Fe, Ni, Cr and Si together with S and P. C, O, Al and Cu are convoluted with contributions from the support grid and holder, based on comparison between multiple precipitates. Spectrum collected with a 45-s dwell

The G phase formed as faceted precipitates of ~ 50 nm equivalent diameter closely associated with the chi phase precipitates, Fig. 7a. When the chi phase precipitate was orientated close to a zone axis, Fig. 7b, the G phase precipitates were orientated on a [110] zone axis (dark contrast in the image). In addition, electron diffraction of the G phase, Fig. 7c, reveals a fcc crystal structure with a lattice parameter of 1.14 nm, with atomic resolution lattice images of this phase are shown in Fig. 7d. High spatial resolution STEM-EDX microanalysis undertaken on thirteen of these nominally G phase precipitates reveal the presence of Fe, Ni, Cr and Si, Fig. 8. The composition corresponds to (Fe,Ni,Mo)_16_(Cr)_6_Si_2_. Previous workers have reported various compositions of the G phase in a range of austenitic stainless steels: a typical example for the compositions is (Ni, Fe, Mo, Cr)_16_(Nb, Mn, Cr, Ti)_6_Si_6 or 7_ [12, 14, 19, 23]. The crystal structure of the G phase detected in the current specimen is consistent with previous observations, although the lattice parameter is slightly smaller (*a* = 1.115–1.120 nm [12]). However, the phase appears to have a significantly lower silicon content compared to other studies [12, 14, 19, 23]. It is possible that this difference accounts for the smaller lattice parameter of the crystal lattice compared to previous studies. In this case, 92% of the G phase precipitates were found on the austenite side of austenite–ferrite and austenite–chi phase boundaries, with the remaining precipitates sited on ferrite–ferrite precipitate boundaries. It has been noted that frequently impurity P is associated with G phase precipitates [40], but it was not unambiguously confirmed for the present precipitates.

#### Intragranular γ′ precipitates

**Figure 9:**
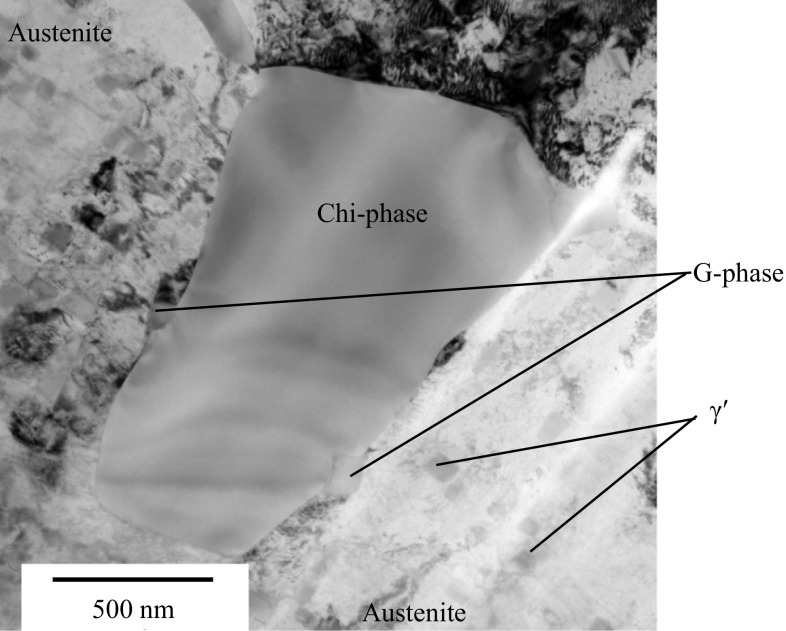
TEM image showing the distribution of precipitates in Cr-rich region of an ex-service + 22000-h aged Type 316H steel

Small cubic intragranular precipitates within the austenite grains were also identified in the Cr-rich regions, Fig. 9.

**Figure 10:**
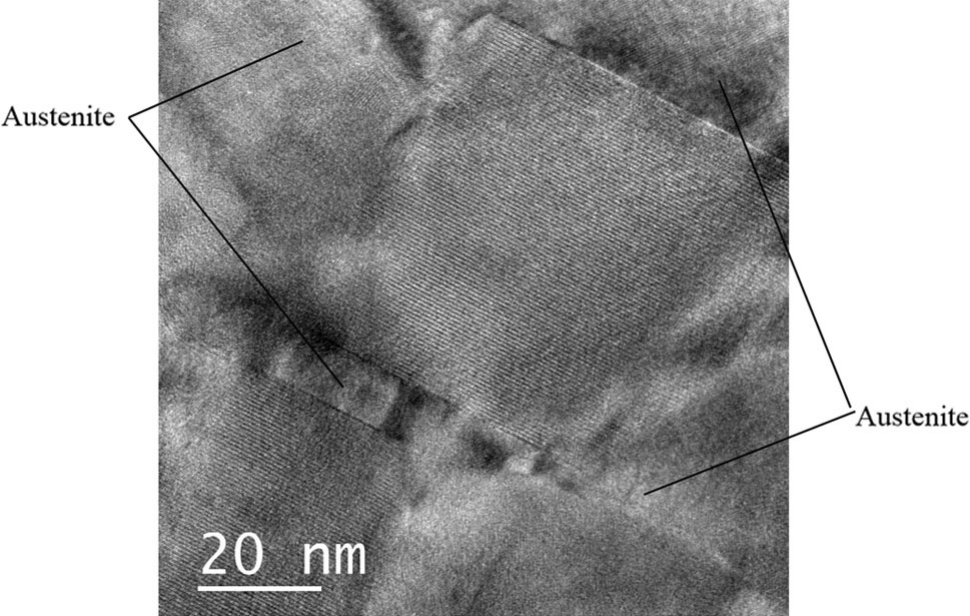
Atomic resolution image of two intragranular γ′ in an austenite matrix in the ex-service + 22000-h aged Type 316H steel

These precipitates typically showed a degree of alignment, consistent with a fixed orientation relationship with the austenite grain. An atomic resolution HRTEM image of two of these ordered precipitates is shown in Fig. 10. Characterising the FFT from one of the precipitates, derived from the HRTEM images, Fig. 10, gives an atomic spacing consistent with a lattice parameter of between 0.331 and 0.337 nm. Microanalysis composition measurements taken from four precipitates were averaged to give the composition in Table 3. This lattice parameter and composition correlates well with γ′.

**Table 3:** Measured average compositions, crystal structures and lattice parameters of the secondary phase precipitates present in the fine-grained, high Cr residual casting regions present in an ex-service + 22000-h aged Type 316H austenitic steel

Phase	Crystal structure	Measured lattice parameter (nm)	Mean measured composition—wt%										
			C	O	Al	Si	S	P	Cr	Mn	Fe	Ni	Mo
Chi phase	BCC	0.795–0.845	–	–	–	–	2 ± 3	–	53 ± 10	–	28 ± 5	3 ± 1	2 ± 2
G phase	FCC	1.115–1.14	–	–	–	5 ± 2	1 ± 2	–	21 ± 12	–	20 ± 15	18 ± 7.6	24 ± 14
Intragranular γ′ precipitates	FCC	0.331–0.337	–	–	–	–	–	–	44 ± 1	–	47 ± 5	8 ± 4	1 ± 0.7

C data excluded due to contamination; O and Al contributions are conflated with contributions from the grid and holder and thus excluded

### Spatial relationships between chi phase, G phase and γ′

Across the majority of the TEM liftouts, it was noted that within the Cr-enriched regions the phases had distinct relationships with each other. Chi phase precipitates were associated with G phase precipitates, although G phase was associated with both chi phase and ferrite precipitates (not shown). The austenite surrounding the chromium-rich chi phase precipitates was depleted of intragranular γ′, Fig. 3, consistent with local depletion of the austenite in chromium.

**Figure 11:**
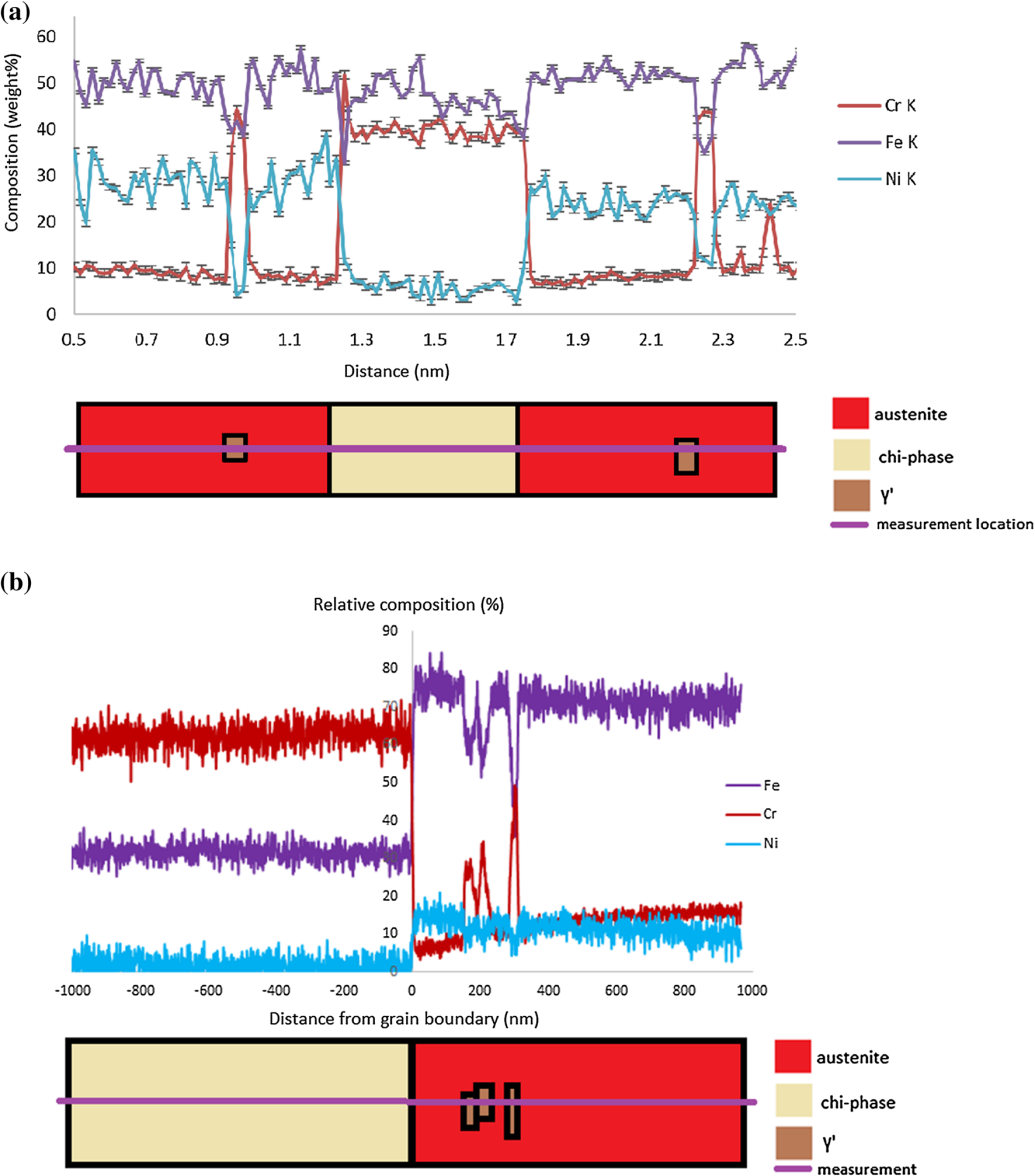
Composition analysis across a chi phase precipitate. **a** Shows an EDX line scan across a chi phase precipitate and **b** EELS line scan across a chi phase precipitate, including several γ′ precipitates in the austenite (denoted by the spikes in Cr). Note the Cr-depleted region in the austenite within 200 nm of the chi phase boundary

The austenite–chi phase grain boundary composition line scan in Fig. 6 shows no difference in Cr distribution in the austenite within 20 nm of the chi phase boundary. However, if the compositional analysis is extended further into the neighbouring grains, Fig. 11a, it becomes clear that the austenite surrounding the chi precipitate is relatively depleted in chromium compared to the bulk of the austenite grain. An electron energy loss spectroscopy (EELS) line scan across the same chi phase precipitate also shows chromium depletion, Fig. 11b.

## Discussion

**Figure 12:**
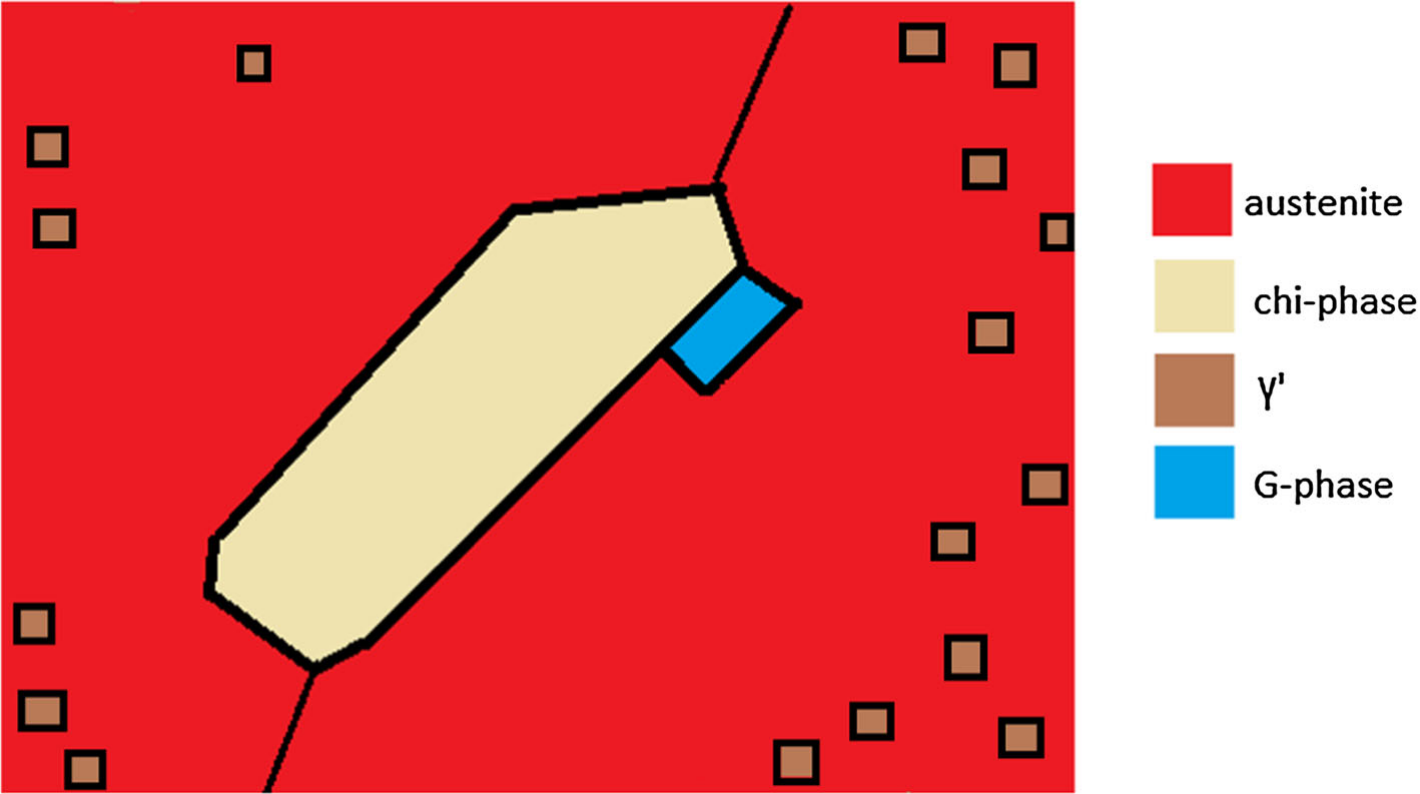
Schematic diagram showing the typical distributions of secondary phases in the localised Cr-enriched regions of an ex-service + 22000 h at 550 °C aged Type 316 steel

The ex-service Type 316H stainless steel has a very complex microstructure arising from the fabrication history which does not introduce sufficient deformation to homogenise the microstructure and therefore leaves local regions of different composition arising from the original cast ingot [5]. TEM liftouts were cut from these high Cr fine-grained regions identified by Chen [41] and Warren et al. [5] reveal that a complex mix of precipitates arises as a consequence of long-term service at temperatures of ~ 550 °C, including α-ferrite, chi phase, γ′ and G phase, as summarised in Tables 2 and 3, and shown schematically in Fig. 12.

The composition of the austenite in the bulk regions is 17.5% Cr and 12.05% Ni whilst the main secondary phase precipitate is M_23_C_6_ carbide [37]. In the localised Cr-enriched residual casting regions, the mean bulk Cr value is 18.0%, Mo is 0.65% and Ni is 9.89%. This significant difference in behaviour can provide an insight into the role some elements play on long-term precipitate evolution.

In general, the current understanding of the precipitation behaviour of secondary phases in austenitic stainless steels is based upon the consideration that the material is homogenous. Few studies consider prolonged ageing at temperatures around 500 °C and the interaction between evolving phases. Weiss and Stickler’s time–temperature–precipitation diagram for aged Type 316H predicts only M_23_C_6_ carbide formation under present ageing conditions [9].

The CALPHAD model developed by Yang and Busby [10] considers a steel of similar composition to that used in this study, with comparable Ni and Si levels. This predicts that within the parent austenite, sigma phase, Laves phase, M_23_C_6_ carbide and an iron-rich ferrite phases precipitate under equilibrium conditions at 550 °C. G phase precipitates are not predicted at any temperature, whilst chi phase formation is predicted only for temperatures that exceed 750 °C and γ′ (Ni_3_Si) formation for temperatures under 400 °C [10]. These predictions differ significantly from the observed phases that precipitated in the localised regions of the present material that are higher in concentrations of Cr. Based on these studies and those discussed in the introduction, the proposed precipitation sequence is:

- (Microstructure as fabricated (AF): Austenite + δ-ferrite + M_23_C_6_)
- → AF + G phase + M_23_C_6_
- → AF + G phase + M_23_C_6_ +”High Fe” α-ferrite [5] + α-ferrite + chi phase
- → AF + G phase + M_23_C_6_ +”High Fe” α-ferrite [5] + α-ferrite + chi phase +γ′

Given the thermo-mechanical history of the samples, the evolution of G phase at austenite grain boundaries would be expected [12, 19]. Previous studies have not shown G phase associated with chi phase precipitates. Due to the history of the material, it is unclear whether the G phase evolved from the chi phase or whether the G phase evolved from a ferrite–precipitate which subsequently transformed into chi phase. Previous studies have noted the compositional similarity between chi phase and sigma phase [12, 26], whilst G phase has been observed in association with sigma phase—as such, this correlation appears reasonable. Likewise, G phase is known to form from ferrite [13, 19] so this also represents a plausible possibility. Further work is required to clarify this. The measured lattice parameter correlates with other observations of G phase (FCC, *a* = 1.115–1.12 nm [12]). If the G phase precipitates in the ex-service steel are normalised for Fe, Ni and Mo, an average measured composition becomes (Fe, Ni, Mo)_16_(Cr)_6_Si_2_. A theoretical composition for G phase has been given as (Ni, Fe, Cr)_16_(Nb, Ti)_6_Si_6_ [12]; however, substitution of several alloying elements has previously been observed such that a more representative combination would be (Ni, Fe, Mo, Cr)_16_(Nb, Mn, Cr, Ti)_6_Si_6 or 7_ [14, 19, 23] or as generalised by Sourmail to be A_16_D_6_C_7_ where A and D are transition elements and C is a group IV element [24]. Although the ratio of alloying elements shown in Table 3 is comparable to that observed in G phase, the silicon level is significantly lower. The ex-service material has a bulk Si content lower than the steels used in many studies on G phase, which is reflected in the low ferrite to austenite Si ratio; ~ 0.6 wt% in ferrite; and ~ 0.8 wt% in austenite. No significant elemental segregation was observed at the austenite–G phase boundary. Based on observations, both the austenite–chi phase and the austenite–ferrite interfaces provide nucleation sites. However, chi phase presents a preferred nucleation site due to the close lattice parameter matching with the nucleating G phase (ferrite *a* = 0.285–0.289 nm, chi phase *a* = 0.795–0.845 nm, austenite *a* = 0.358 and G phase *a* = 1.115–1.12 nm).

Chi phase precipitates were frequently observed and were typically associated with G phase precipitates. It is unclear at what stage of the thermal ageing the chi phase precipitates in the material evolved—the closest comparable literature observation to the specimen (Type 316 N steel/85000 h creep test/550 °C) [42] suggests that in the present case the chi phase precipitates would have formed during the additional 22000 h post-service ageing. However, given that chi phase is typically formed in the high Cr δ-ferrite rich regions, duplex steels or Type 300 series weld metal may provide a better basis for comparison. Assuming this to be the case, there have been observations of chi phase after only 5000 h at 500 °C [31] and rapid chi phase precipitation at 600 °C [30, 34, 43] suggests that evolution will have occurred during the service life of the material. Hence, the initial nucleation of chi phase occurs during service; however, further precipitates may nucleate and grow during the post-service heat treatment. Studies have frequently observed the transformation of δ-ferrite to chi phase [26, 29, 31, 32] and as such chi phase precipitates may be considered to mark the sites of former ferrite precipitates.

The observed P enrichment at the austenite–chi phase boundary is consistent with studies by Chen [41] showing the presence of P on low-temperature fracture surfaces. This enrichment was attributed to the segregation to the austenite–ferrite interphase boundary [41]; however, the current work shows that it is the austenite–chi phase boundaries that are enriched in P. The favourability of interphase boundary equilibrium segregation can be assessed by using the free energy of segregation of phosphorus $\Delta G_{P}$, which can be calculated in austenite and chi phase using the equation:

3 $$ \Delta G_{\text{P}} = \Delta H_{\text{P}} - T\Delta S_{\text{P}} $$

where Δ*H*_P_ is the standard molar enthalpy of phosphorus segregation, *T* is the temperature and Δ*S*_P_ is the standard molar entropy of phosphorus segregation. Ševc et al. [44] determined the Δ*H*_P_ and Δ*S*_P_ of phosphorus in a Type 304 as 14.1 ± 8.3 kJ/mol and 15.0 ± 8.5 J/(mol k), respectively. These data correlate well with the Paju and Grabke measurements of segregation in a Fe–P binary alloy at austenite temperatures [45]. Δ*H*_P_ and Δ*S*_P_ for phosphorus segregation in chi phase are not known, and as such the values for segregation in ferrite have been used as a first approximation. These were determined as − 34.3 kJ/mol and 21.5 J/mol, respectively [46, 47]. Using these values in Eq. 3, it is possible to calculate the free energy of segregation of P in austenite as -25.7 kJ/mol and in ferrite (approximating chi phase) as − 51.0 kJ/mol. P segregation to the austenite–chi phase boundary, Fig. 5, will be more favourable than that predicted for an equivalent austenite–ferrite boundary as the greater lattice mismatch between the phases will provide more favourable sites for P (chi phase *a* = 0.795–0.845 nm, austenite *a* = 0.358 nm and ferrite *a* = 0.285–0.289 nm).

Using the modified Langmuir–McLean formalism and considering site competition [44, 46] together with the measured compositions for austenite-chi phase grain boundaries, it is possible to calculate the free energy of segregation for phosphorus, Δ*G*_P_, at an austenite–chi phase boundary:

4 $$ \frac{{X_{\text{P}} }}{{1 - \varSigma_{{{\text{J}} \ne {\text{Fe}}}} X_{\text{J}} }} = \frac{{X_{\text{P}}^{\text{B}} }}{{1 - \varSigma_{{{\text{J}} \ne {\text{Fe}}}} X_{\text{J}}^{\text{B}} }}\exp \left( { - \frac{{\Delta G_{\text{P}} }}{RT}} \right) $$

where $X_{\text{P}}$ is the concentration of P at the boundary in mols, $X_{\text{P}}^{\text{B}}$ is the concentration of P in the bulk, $X_{\text{J}}$ the concentration of an element and the boundary (J = P, Cr, Ni) and $X_{\text{J}}^{\text{B}}$ the concentration of the element in the bulk. *R* is the gas constant and *T* the absolute temperature. Taking the composition at the boundary, and the mean composition from austenite and chi phase as a bulk composition for the ageing conditions present during service, $\Delta G_{\text{P}}$ = −101.5 kJ/mol. Hence, the segregation of phosphorus at the austenite–chi phase boundary is significantly more favourable during service than segregation to either austenite–austenite or chi phase–chi phase boundaries, correlating with the measured component and distribution of the phosphorus.

The lattice spacings extracted from the FFT for the intragranular precipitates correlate well with γ′ precipitates [12]. Frequently, γ′ precipitates are observed in superalloys such as Inconel alloys, with compositions of (Ni, Co, Fe, Cr)_3_(Al, Ti) [12, 48]. In Type 316 steels, it has been observed following irradiation and thermal ageing in the temperature range 270–540 °C with a composition of Ni_3_(Si, Al, Ti, Nb), and a high solubility of the elements Fe, Cr and Mo [48–50]. It has been proposed that neutron irradiation has a critical role in γ′ formation, or at least rapidly enhanced the formation kinetics [48]. The γ′ precipitates in current material do not contain Al or Ti. It is suggested that Si can act as an ionic substitute for Ti, in which case it is possible that the low volume of Si in the precipitates fulfils the role of Ti. Given the high quality of atomic resolution images recorded for the γ′ precipitates, it is unlikely that the measured compositions are influenced by the presence of austenite.

The chi phase precipitates were surrounded by a chromium-depleted region, Fig. 12, and this region contained no γ′ precipitates. This depleted austenite region as a minimum contains ~ 6.5 wt% Cr, rising to ~ 10 wt% in the surrounding austenite, Fig. 12. γ′ phase precipitates in regions of austenite where the Cr concentration is greater than 8.3 wt%. Given the partial uncertainty regarding the precipitation sequence, it is not known if γ′ precipitates are taken into solution during the growth of the chi phase or are prevented from forming due to the presence of a chromium-depleted zone. Regardless, it suggests that γ′ is highly sensitive to Cr concentration and only exists due to the abnormally high levels of Cr in these local regions of the overall microstructure.

There has been a significant evolution of the microstructure between the solution-treated pre-service specimen, and the extensively aged post-service specimens. Given that the presence of ferrite precipitates has previously been shown to effect the creep cavitation behaviour of austenitic steels [5], it would be expected that the wide range of precipitates observed in high Cr local regions in the post-service specimen modify the creep behaviour of the material, and this will be considered in a future paper. Certainly a distribution of accelerated cavitation that link to form localised creep-induced cracks has the potential to degrade overall creep life.

## Conclusions

An ex-service Type 316H component was observed to have localised regions with different microstructure within the bulk material after prolonged service followed by laboratory exposure at a temperature of ~ 550 °C. These regions contain a greater number of ferrite precipitates and a finer austenite grain structure than the majority of the material. Previous workers have detected a higher concentration of Cr and impurity elements in these regions and have characterised them as heterogeneity arising from the original cast microstructure [5]. The present studies show that these regions have a highly complex microstructure containing G phase, chi phase and γ′ precipitates within the austenite matrix.

- Faceted G phase precipitates with a composition of (Fe, Ni, Mo)_16_(Cr)_6_Si_2.3_ have been observed at austenite–chi phase boundaries. Phase identification has been achieved using fast Fourier transform-generated diffraction patterns from atomic resolution images of the phase. This G phase has a FCC crystal structure together with a lattice parameter of 1.00 nm. The G phase precipitates have been observed to have a very strong orientation relationship with the neighbouring chi phase, based on observations made during tilt analysis.
- Chi phase precipitates were observed at austenite–austenite boundaries, showing a faceted morphology and equilibrium P segregation at the austenite–chi phase boundary.
- Intragranular Fe/Cr-based γ′ precipitates were observed in the austenite, and these have a lower concentration of Si than previous observations.
- A Cr-depleted region was measured in the austenite matrix surrounding chi phase precipitates, where otherwise ubiquitous γ′ precipitates were absent; either γ′ formation is inhibited or the growing chi phase causes dissolution of γ′ precipitates due to competition for Cr.
